# Nasopharyngeal carriage of *Streptococcus pneumoniae,* its associated factors, and antimicrobial susceptibility patterns among school children in Babile district, eastern Ethiopia

**DOI:** 10.1371/journal.pone.0337950

**Published:** 2025-12-05

**Authors:** Fuad Abdi Mohammed, Rajesh Sarkar, Firayad Ayele, Kedir Urgesa

**Affiliations:** 1 Bisidimo General Hospital, Bisidimo, Eastern, Ethiopia; 2 School of Medical Laboratory Sciences, Haramaya University College of Health and Medical Sciences, Harar, Ethiopia; Mekdela Amba University, ETHIOPIA

## Abstract

**Background:**

*Streptococcus pneumoniae* colonization is a growing concern globally, including in Ethiopia. School-aged children are a key reservoir that can lead to endogenous infections and a potential source for the transmission in the community. However, little is known about its nasopharyngeal carriage rates in eastern Ethiopia. This study aimed to determine the nasopharyngeal carriage of *Streptococcus pneumoniae*, associated factors, and antimicrobial susceptibility patterns among primary school children in Babile District, Eastern Ethiopia.

**Methods:**

A cross-sectional study was conducted at primary schools in the Babile district, Eastern Ethiopia, from November 15, 2022, to January 8, 2023. A systematic random sampling technique was used to enroll 337 schoolchildren. Data on sociodemographic and a potenail associated factors was collected using structured questionnaires. Nasopharyngeal swab samples were collected, transported in Amies medium, and cultured on blood and chocolate agar. *Streptococcus pneumoniae* was identified based on colony morphology, Gram staining, hemolysis, and biochemical tests. Antimicrobial susceptibility testing was performed using the Kirby-Bauer disk diffusion method. Data was entered in EpiData and analyzed in SPSS. Bivariate and multivariable logistic regression was used to identify factors associated with pneumococcal carriage, with statistical significance set at p < 0.05 and 95% CI.

**Results:**

Overall nasopharyngeal carriage of *Streptococcus pneumoniae* was 16% (54/337) (95% CI: 12.0–20.0). The ages of the children ranged from 7 to 17 years, with a mean age of 12.75 years (SD ± 2.56). Passive smoking (AOR = 2.86, 95% CI: 1.45–5.67), single room house (AOR = 2.69, 95% CI: 1.32–5.49), greater than or equal to two siblings under 5 years old in the house(AOR = 4.8, 95% CI: 1.88–12.25), and previous respiratory tract infection (AOR = 3.24, 95% CI: 1.66–6.32) were significantly associated with nasopharyngeal carriage of *Streptococcus pneumoniae.* The isolated *Streptococcus pneumoniae* showed higher drug resistance to Tetracycline 23 (42.6%) and Trimethoprim-sulfamethoxazole (TMP-SMX) 18 (33.3%) and was found to be highly susceptible to oxacillin 32(72.2%), Vancomycin 41(75.9%) and Erythromycin 44 (81.48%).

**Conclusion:**

A considerable proportion of asymptomatic nasopharyngeal carriage of *Streptococcus pneumoniae* in school children was associated with having a history of respiratory tract infection, being passive smokers, having greater than or equal to two siblings under 5 years old in the house, and living in a single-room house. A higher resistance of isolated *Streptococcus pneumoniae* was observed to tetracycline and trimethoprim-sulfamethoxazole. Thus, it is necessary to regularly assess the trend of antibiotic resistance andthe prevalence of pneumonia among asymptomatic children, and it is impretive to focus on modifiable associated factors in controlling the diseases.

## Introduction

*Streptococcus pneumoniae* is a gram-positive, extracellular pathogen that is the leading cause of bacterial pneumonia. It is also the primary cause of death from infectious diseases among children worldwide [1]. In addition to pneumonia, *S. pneumoniae* can also lead to serious conditions such as sepsis, meningitis, sinusitis, and acute otitis media [2–4]. *S. pneumoniae* is also a prevalent component of the nasopharyngeal flora in healthy children [5,6].

According to the World Health Organization, approximately 1 million young children die each year due to pneumococcal disease [7], with one child under five years old dying from it every 20 seconds. Around 50% of these total pneumonia deaths occur in just six countries, including Ethiopia [8]. In the United States, it’s estimated that there are around 40,000 pneumococcal infections annually [9]. Similarly, each year, Africa reports around one to four million cases of pneumonia in children. In Ethiopia, Streptococcus pneumoniae is responsible for 21.4% of the severe cases. [10]. Across sub-Saharan Africa, *S. pneumoniae* accounts for 25–30% of meningitis cases and 30–50% of pneumonia cases in children under 5 years old [11]. Pneumococcal colonization is most common in the first few years of life, peaking at 50–80% in 2–3 year olds before declining to 5–10% in children older than 10 [12].

Nasopharyngeal colonization is a precondition for pneumococcal disease progression and a major source of horizontal transmission in the population, particularly in highly populated environments such as schools [13,14]. Young children are the most important reservoir for the transmission of pneumococcal infections since their colonization is higher [15]. *S. pneumoniae* occurs primarily by indirect contact *via* inhalation of airborne droplets [16,17]. Increases in *S. pneumoniae* colonization have been associated with factors such as young age, crowding, family size, number of siblings, poverty, smoking, and recent antibiotic usage [18].

The increasing prevalence of antimicrobial-resistant pneumococcal strains is a global health concern, particularly in Ethiopia [19,20], where *Streptococcus pneumoniae* mortality among children under five remains high despite the introduction of the Pneumococcal conjugate vaccine [21]. Antimicrobial resistance is exacerbated by the high rates of nasopharyngeal colonization, especially in crowded school settings, which facilitates the dissemination of resistant strains [22,23]. However, data on nasopharyngeal carriage and antimicrobial-resistant *S. pneumoniae* in Ethiopia are limited. This study aimed to determine the nasopharyngeal carriage of *Streptococcus pneumoniae*, associated factors, and antimicrobial susceptibility patterns among primary school children in Bisidimo, Babile District, Eastern Ethiopia.

## Materials and methods

### Study area, design, and period

This study was conducted in Bisidimo, located in the Babile district of the Oromia region in Eastern Ethiopia. A school-based cross-sectional study design was employed. Bisidimo is notable for its unique community structure, as it is situated in a leprosarium area. The town is 546 km from Addis Ababa, the capital city of Ethiopia. The Babile district comprises 42 schools, including 3 preparatory, 5 secondary, and 34 primary schools. In Bisidimo specifically, there is 1 preparatory school, 1 secondary school, and 8 primary schools (Esakoy, Abbeye, Kufakasa, Nejata, Ererebada, Biftu, Efadin, and Ebroseden). The study was conducted from November 15, 2022, to January 8, 2023.

### Population, inclusion, and exclusion criteria

The source population for this study consisted of all school children attending primary schools in Bisidimo during the study period. The study population was narrowed down to school children attending randomly selected primary schools in Bisidimo during the same time frame. The inclusion criteria were children attending the selected primary schools, aged 7–17, and with parental consent to participate. Exclusion criteria were children with signs or symptoms of respiratory tract infection or who had used antibiotics within the 3 months prior to data collection.

### Sample size determination and sampling technique

**Fig 1:**
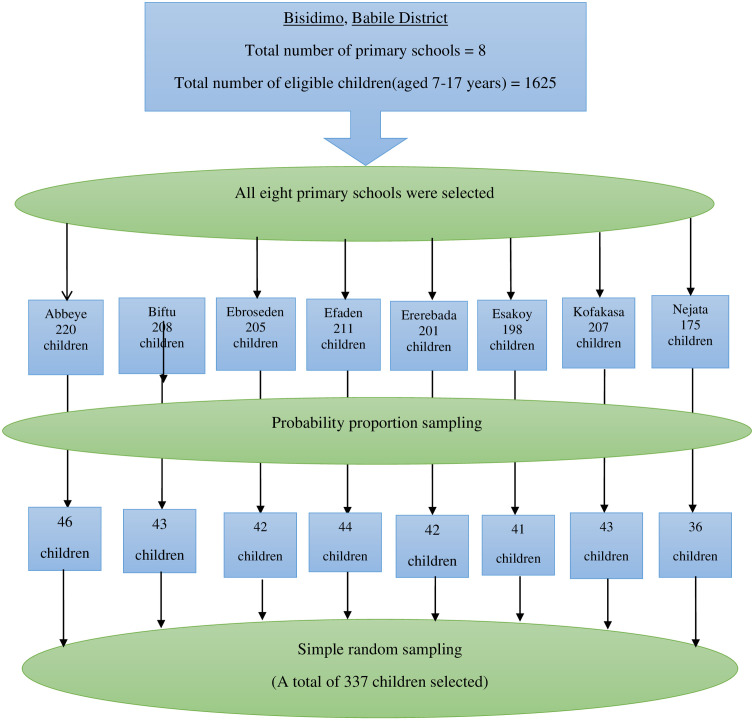
Sampling technique among primary school children aged 7-17 years in Bisidimo, Babile district, Eastern Ethiopia.

The sample size was determined using the formula for a single population proportion, n = Z^2 * p(1-p)/ d^2, where n is the sample size, Z is the reliability coefficient (95% = 1.96), p is the expected population proportion (43.8% = 0.438) [23], and d is the margin of error (5% = 0.05), which resulted in a calculated sample size of 378; however, since the source population (1,625) was less than 10,000, the sample size was recalculated using the correction factor formula, final sample size = n/ (1 + n/N), where N is the source population, resulting in a final calculated sample size of 306. All eight primary schools in the Bisidimo area (Abbeye, Biftu, Ebroseden, Efadin, Ererebada, Esakoy, Kufakasa, and Nejata) were included in the study, and the total sample size was allocated proportionally to each school and then equally distributed across all eight grades (1–8); one section was randomly selected from each grade, the student name lists were obtained, and the final study participants were selected from these section lists using simple random sampling techniques until the full sample size of 306 was reached (Fig 1).

### Data and sample collection

The questionnaire was prepared in English, then translated into the local language (Afan Oromo), and then re-translated back to English to keep the reliability of data collection. Structured questionnaires were used to collect data on socio-demographic and associated factors such as age of the child, sex of the child, parents’ educational status, religion, occupation, family size, presence of <5 years siblings, presence of siblings ≥5 years, number of rooms in a house, the habit of sleeping with parents, previous antibiotic use, previous hospitalization, and respiratory tract infections. Parents of children attending selected schools consented. After written informed consent was obtained from the parents, they were interviewed for socio-demographic characteristics, previous health conditions of their children, and related associated factors of *S. pneumoniae* by five trained laboratory technicians under two supervisors.

After written informed consent was obtained from the parents, the nasopharyngeal specimen was collected by a trained laboratory technician. The technicians collected the specimen by using a cotton-tipped flexible swab. A specimen was collected by passing a sterile cotton-tipped flexible swab gently through the nostril along the nasal cavity until it touched the posterior wall of the nasopharynx. Once the swab touched the posterior wall of the nasopharynx, it was rotated and left there for 5 seconds to saturate the tip [24]. After being collected, the swab specimens were placed in Amie’s transport medium and transported to the Hiwot Fana Comprehensive and Specialized University Hospital microbiology laboratory within one hour of collection.

### Bacterial isolation and identification

A nasopharyngeal specimen was inoculated onto chocolate agar and 5% sheep blood agar, then incubated for 24 hours at 35°C with 5% CO_2_. *Streptococcus pneumoniae* was identified based on colony morphology (small, grayish, mucoid colonies), gram staining (gram-positive diplococci), alpha-hemolysis on blood agar, optochin susceptibility (zone of inhibition ≥14 mm), and bile solubility (lysis upon bile salt addition). Additionally, a catalase test confirmed the isolate as catalase-negative, corroborating its classification as a member of the Streptococcus genus [25].

#### Antimicrobial susceptibility testing.

The antimicrobial susceptibility pattern of *Streptococcus pneumoniae* isolates was evaluated using the disk diffusion method on Mueller-Hinton agar (MHA) supplemented with 5% sheep blood, as organism is fastidious nature. An overnight colony suspension was prepared in 0.85% NaCl to achieve the 0.5 McFarland standard, which was then inoculated onto the MHA plates and incubated in a 5% CO2 environment for 18–24 hours [26]. Conventional identification of *S.pneumoniae* and antimicrobial susceptibility testing [18] was done according to appropriate standards, and erythromycin (15 μg), vancomycin (30 μg), clindamycin (2 μg), tetracycline (30 μg), chloramphenicol (30 μg), oxacillin (1 μg) and trimethoprim sulphamethoxazole (25 μg) were used for AST [26].

#### Data quality control.

To ensure consistency, the questionnaire was first drafted in English, then translated into Afan Oromo, and subsequently translated back into English by a language expert. Data collectors underwent comprehensive training on sample collection, transportation, and processing before the data collection commenced. A pretest was administered to 5% of primary students in Harar who were not part of the main study, with adjustments made based on the pretest findings. Daily, the principal investigator and data collectors reviewed all completed questionnaires for completeness, clarity, and consistency. Data quality was upheld through standardized collection materials, effective training, and diligent supervision during the data collection process. For laboratory analysis, strict adherence to quality assurance protocols for the pre-analytical, analytical, and post-analytical stages was maintained, following the standard operating procedures (SOPs) of the microbiology laboratory. Quality control was conducted using known controls.All samples collected were cultured immediately after collection. Rejection criteria applied to those which were deemed unfit for processing, such as mislabeled specimens. Five percent (5%) of the prepared culture Media was randomly selected and incubated aerobically for 24h. at 35^0^C to check the sterility of the prepared culture media. *S. pneumoniae* American Type Culture Collection (ATCC 49,619) [26] was used as a positive control strain on each procedure. Laboratory identification procedures like inoculation of culture media, colony characterization, and measuring of drug susceptibility testing were checked by an experienced microbiologist.

#### Operational definitions.

**Socioeconomic status:** is a measure of an individual’s or group’s economic and social position in relation to others, based on factors such as income, education, and occupation [27].

**Previous antibiotic use:** consumption of any antibiotics in the 3 months prior to the date of data collection [28].

**Nasopharyngeal carriage**: the presence of bacteria in the nasopharynx without causing symptomatic disease [29].

**School children:** Primary school childen in age rage from 7 to 17.

### Data analysis and interpretations

Data were entered into EpiData version 3.1, a software designed for accurate data entry and validation, before being exported to SPSS version 25 for analysis. Descriptive statistics were employed to summarize key aspects, including socio-demographic characteristics, carriage rates of Streptococcus pneumoniae, and susceptibility patterns of isolates. Prior to analysis, the data collection process involved rigorous checks for completeness and consistency, with data collectors trained to meticulously record responses. Bivariable logistic regression was initially used to explore relationships between individual risk factors and the nasopharyngeal carriage rate, identifying variables with p-values less than 0.25 for inclusion in the multivariable analysis. This analysis was conducted using the forward selection method, allowing for the systematic inclusion of statistically significant variables. To ensure robustness, multicollinearity among independent variables was assessed using the variance inflation factor (VIF), with values above 10 indicating potential issues. The fit of the logistic regression model was evaluated with the Hosmer–Lemeshow goodness-of-fit test, comparing observed and expected frequencies. In the final multivariable analysis, variables with p-values less than 0.05 were deemed statistically significant, highlighting key factors associated with S. pneumoniae carriage. Overall, this study aimed to provide insights into significant associations that could inform public health strategies for managing S. pneumoniae carriage among school children.

Data were entered into EpiData version 3.1 and then exported to SPSS version 25 for analysis. Descriptive statistics summarized the socio-demographic, carriage rate, and susceptibility patterns of isolates. Data was collected and checked for completeness and consistency before analysis. Bivariable and multivariable logistic regression analyses were carried out to identify potential association factors of the nasopharyngeal carriage rate of *S. pneumoniae* amongst the school children. Adjusted odds ratio with the corresponding 95% confidence intervals [30] was used to measure the association between risk factors and nasopharyngeal carriage. All variables with p-values less than 0.25 during bivariate analysis were candidates for Multivariable analysis.Multivariable logistic regressionwas performed using the forward method. Multicollinearitywas checked using the variance inflation factor. The model fit-ness was checked using the Hosmer–Lemeshow goodness-of-fittest which was fitted. All variables with p-values less than 0.05 during multivariable analysis were considered statistically significant.

### Ethical consideration

Written ethical clearance was obtained from the Institutional Health Research and Ethical Review Committee (Ref. No. IHRERC/183/2022) of the College of Health and Medical Sciences (CHMS), Haramaya University. A permission letter was obtained from Haramaya University, and the information that was provided was submitted to the respective schools. Before data collection, both the student’s parents and the students are informed about the study’s procedures, including potential risks, benefits, and confidentiality. The students agree to participate with their parents signing an assent form, while the parents provide informed consent for their child’s involvement. Moreover, the local health institution and Bisidimo Hospital administration were informed about the conditions of those children who were confirmed positive and their treatments through intervention in collaboration.

## Result

### Socio-demographic characteristics

In this study, a total of 337 children participated with a response rate of 100%. The age range of the children was 7–17, with a mean age of 12.75 ± SD 2.56. More than half, 185 (54.9%) were male and nearly half (43.9%) of participants mothers didn’t attend formal education. Most (73%) students’ mothers were housewives and 68.2% of their fathers were farmers. The majority (90.5%) of participants’ parents/guardians were married, and 62.6% of them got less than 1000 Ethiopian birr income per month. Morethan half(53.1%) of participants were living in a single-room house. More than 2/3(66.2%) of participants had never owned a separate kitchen (Table 1).

**Table 1:** Socio-demographic characteristics of children who attended primary schools in Bisidimo, Babile District, East Ethiopia, (N = 337).

Variables	Category	Frequency (N)	Percents (N%)
Age (in years)	7-10	62	18.4
	11-14	181	53.7
	15-17	94	27.9
Gender	Male	185	54.9
	Female	152	45.1
Religion	Orthodox	60	17.8
	Muslim	245	72.7
	Protestant	12	3.6
	Others	20	5.9
The educational status of the father	Did not attend formal education	85	25.3
	Primary school	140	41.5
	Secondary school	73	21.7
	College/University	39	11.6
Educational status of the mother	Did not attend formal education	148	43.9
	Primary school	125	37.1
	Secondary school	39	11.6
	College/University	25	7.4
The occupational status of the father	Government employee	57	16.9
	Farmer	226	67.1
	Labor worker	29	8.6
	Merchant	21	6.2
	Others	4	1.2
Occupational status of the mother	Government employee	43	12.8
	Housewife	239	70.9
	Labor worker	14	4.2
	Merchant	36	10.7
	Others	5	1.5
Marital status of parents/guardians	Single	5	1.5
	Married	305	90.5
	Divorced	18	5.3
	Widowed	9	2.7
Monthly income (ETB) of parents/guardians	<1000	211	62.6
	1001-2000	65	19.3
	2001-3000	20	5.9
	>3000	41	12.2
Number of families in the house	< 5 members	44	13.1
	≥ 5 members	293	86.9
Number of siblings < 5 years old in the house	0	91	27
	1	120	35.6
	≥ 2	126	37.4
Number of siblings ≥ 5 years old in the house	0	14	4.2
	1	31	9.2
	≥2	292	86.6
Smoker in the house	yes	159	47.2
	No	178	52.8
Sharing bed with parents	yes	179	53.1
	No	158	46.9
Cooking in the bedroom	Yes	114	33.8
	No	223	66.2
Number of rooms per house	1	179	53.1
	≥2	158	46.9

### Clinical characteristics of study participants

The majority (92.9%) of participants had taken the pneumococcal conjugate vaccine, as checked by their PCV vaccination schedule card. About 39.8% of participants had a history of respiratory tract infections in the last three months (Table 2).

**Table 2:** Clinical characteristics of children attending primary schools in Bisidimo, Babile District, East Ethiopia (N = 337).

Variables	Category	Frequency (N)	Percents (N%)
Respiratory tract infection s in the last 3 months	yes	134	39.8
	No	203	60.2
Hospitalization history of the last 3 months	yes	22	6.5
	No	315	93.5
Antibiotic use in the last three months	Yes	21	6.2
	No	316	93.8
PCV vaccination status	Vaccinated	313	92.9
	Not vaccinated	24	7.1

### Prevalence of nasopharyngeal carriage of *S. pneumoniae*

The overall prevalence of nasopharyngeal carriage of *S. pneumoniae* among study participants was 16% (54/337) (95%, CI: 12.0–20.0). The highest carriage rate 36 (66.7%) of *S. pneumoniae*, was observed among students aged 11–14 years old. The majority of *S. pneumoniae* carrier children 33(61.1%) were from low socioeconomic classes less than 1000 ETB per month. Of the isolated carriers, more than half 49(90.7%) children were from a family with more than five members. S. *pneumoniae* carriage was more common in children living in one room of the house 39(72.2%) (Table 3).

**Table 3:** Bivariate and multivariable analysis of risk factors associated with *S. pneumoniae* among children attending primary schools in Bisidimo, Babile District, East Ethiopia.

Variables	Category	*S.pneumoniae*		Bivariate analysis		Multivariable analysis	
		Pos N(%)	Neg N (%)	COR (95% Cl)	P- value	AOR (95% Cl)	P-value
Gender	Male	33(17.8)	152(82.2)	1.35(0.74-2.45)	0.318		
	Female	21(13.8)	131(86.2)	1			
Age (in years)	7-10	7(11.3)	55(88.7)	0.96(0.35-2.63)	0.937	0.94(0.31-2.85)	0.921
	11-14	36(19.9)	145(80.1)	1.87(0.91-3.88)	0.091	1.99(0.89-4.44)	0.090
	15-17	11(11.7)	83(88.3)	1			
Educational status of the father	Did not attend formal education	13(15.3)	72(84.7)	1.58(0.48-4.19)	0.452		
	Primary school	23(17)	112(83)	1.79(0.58-5.55)	0.308		
	Secondary school	14(17.9)	64(82.1)	1.91(0.58-6.26)	0.283		
	College/University	4(10.3)	35(89.7)	1			
Educational status of the mother	Did not attend formal education	26(17.6)	122(82.4)	1.56(0.44-5.61)	0.494		
	Primary school	21(16.8)	104(83.2)	1.48(0.41-5.40)	0.552		
	Secondary school	4(10.3)	35(89.7)	0.84(0.17-4.11)	0.828		
	College/University	3(12)	22(88)	1			
The occupational status of the father	Government employee	7(12.3)	50(87.7)	1			
	Farmer	38(16.8)	188(83.2)	1.44(0.61-3.43)	0.405		
	Labor worker	5(17.2)	24(82.8)	1.49(0.43-5.18)	0.532		
	Merchant	3(14.3)	18(85.7)	1.19(0.28-5.11)	0.814		
	Others	1(25)	3(75)	2.38(0.22-26.18)	0.478		
Occupational status of the mother	Government employee	5(11.6)	38(88.4)	1			
	Housewife	44(18.4)	195(81.6)	1.72(0.64-4.61)	0.285		
	Labor worker	2(14.3)	12(85.7)	1.27(0.22-7.39)	0.793		
	Merchant	2(5.6)	34(94.4)	0.45(0.08-2.46)	0.354		
	Others	1(2O)	4(80)	1.9(0.17-20.56)	0.597		
Marital status of parents/guardians	Single	1(20)	4(80)	0.87(0.06-12.98)	0.923		
	Married	48(15.7)	257(84.3)	0.65(0.13-3.24)	0.603		
	Divorced	3(16.7)	15(83.3)	0.7(0.09-5.18)	0.727		
	Widowed	2(22.2)	7(77.8)	1			
Monthly income (ETB) of parents/guardians	<1000	33(15.7)	177(84.3)	1.45(0.53-3.96)	0.464		
	1001-2000	12(18.8)	52(81.2)	1.8(0.59-5.53)	0.305		
	2001-3000	4(21.1)	15(78.9)	2.1(0.49-8.81)	0.320		
	>3000	5(11.4)	39(88.6)	1			
Number of families in the house	< 5 members	5(11.4)	39(88.6)	1			
	≥ 5 members	49(16.7)	244(83.3)	1.57(0.59-4.18)	0.370		
Number of siblings < 5 years old in the house	0	8(8.8)	83(91.2)	1			
	1	19(15.8)	101(84.2)	1.95(0.81-4.68)	0.134	2.42(0.93-6.24)	0.068
	≥ 2	27(21.4)	99(78.6)	2.83(1.22-6.56)	0.015	4.8(1.88-12.25)	0.001
Number of siblings ≥ 5 years old in the house	0	1(7.1)	13(92.9)	1			
	1	4(12.9)	27(87.1)	1.92(0.19-19	0.575		
	≥2	49(16.8)	243(83.2)	2.62(0.34-20.51)	0.358		
Smoker in the house	Yes	39(24.5)	120(75.5)	3.53(1.86-6.70)	0.000	2.86(1.45-5.67)	0.002
	No	15(8.4)	163(91.6)	1			
Sharing bed with parents	Yes	32(17.9)	147(82.1)	1.35(0.75-2.43)	0.325		
	No	22(13.9)	136(86.1)	1			
Cooking in the bedroom	Yes	25(21.9)	89(78.1)	1.88(1.04-3.39)	0.036	1.59(0.82-3.11)	0.171
	No	29(13)	194(87)	1			
Number of rooms per house	1	39(21.8)	140(78.2)	2.66(1.40-5.03)	0.003	2.69(1.32-5.49)	0.006
	≥2	15(9.5)	143(90.5)	1			
Respiratory tract infection	yes	34(25.4)	100(74.6)	3.11(1.70-5.69)	0.000	3.24(1.66-6.32)	0.001
	No	20(9.9)	183(90.1)	1			
Previous hospitalization	Yes	7(31.8)	15(68.2)	2.66(1.03-6.87)	0.043	1.99(0.68-5.84)	0.209
	No	47(14.9)	268(85.1)	1			
Previous antibiotic use	Yes	5(23.8)	16(76.2)	1.7(0.59-4.86)	0.320		
	No	49(15.5)	267(84.5)	1			
PCV vaccination status	Vaccinated	49(15.7)	264(84.3)	1			
	Not vaccinated	5(20.8)	19(79.2)	1.41(0.50-3.97)	0.507		

**Notes: 1 = reference.**

### Risk factors associated with nasopharyngeal carriage of S. *pneumoniae*

In bivariate logistic regression analysis, the number of siblings less than five years old in the house, presence of smoker (passive smoker child), number of rooms in the house, previous respiratory tract infection, age groups, cooking in the bedroom, and previous hospitalization were showed significant association as a p-value of **<** 0.25 and were considered as candidate for multivariable logistic regression analysis. In multivariable logistic regression analysis, two or more than two siblings less than five years old in the house, passive smokers, number of rooms in the house, and previous respiratory tract infection were significantly associated with nasopharyngeal carriage of *S. pneumoniae* as a p-value **<** 0.05.

After adjusting for confounding factors, the presence of two or more than two number of siblings less than five years old in the house was almost five times more likely to be *S. pneumoniae* carriers compared to the absence of siblings less than five years old in the house (AOR = 4.8, 95% CI: 1.88–12.25). Passive smokers child were twice more likely to have *S. pneumoniae* in their nasopharynx compared to nonpassive smokers (AOR = 2.86 95% CI: 1.45–5.67). Those children who live in a single-room house were two times more likely to be *S. pneumoniae* culture positive compared to children who live in a house with more than two rooms (AOR = 2.69,95% CI: 1.32–5.49). Children who had a history of previous respiratory tract infection were three times more likely to be *S. pneumoniae* carriers compared to children who had no history of respiratory tract infection (AOR = 3.24 95% CI: 1.66–6.32) (Table 3).

### Antimicrobial susceptibility testing of *S. pneumoniae* isolated

**Fig 2:**
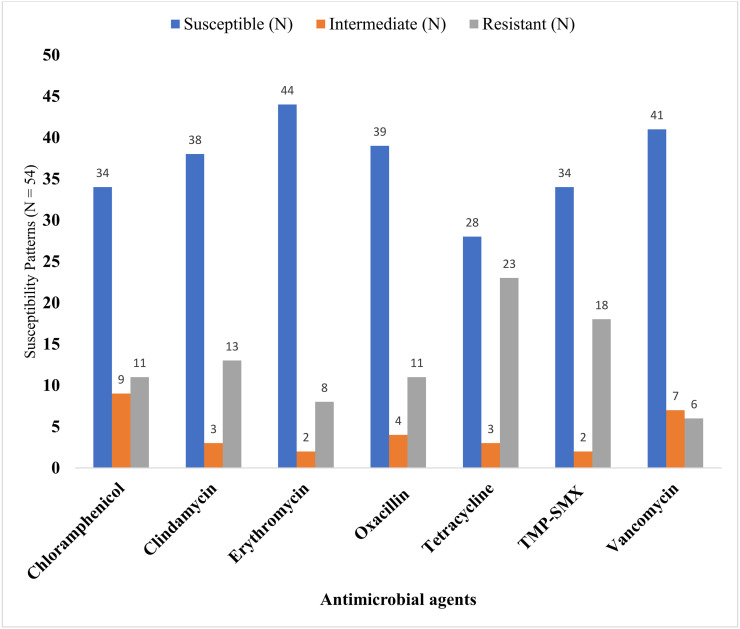
Antimicrobiol Susceptibility patterns of *S. pneumoniae* isolated among children attending primary schools in Bisidimo Eastern Ethiopia 2023.

Antimicrobial susceptibility testing was done for all 54 *S. pneumoniae* isolates to seven antimicrobial agents. In this study, the majority of *S. pneumoniae* isolates were susceptible to erythromycin (81.48%), oxacillin (72.2%), and vancomycin (75.9%). Some of the *S. pneumoniae* isolates showed a higher degree of resistance to tetracycline (42.6%), TMP-SMX (33.3%) and clindamycin (24.07%) comparing to the other antimicrobial drugs used (Fig 2).

## Discussion

Schoolchildren carrying *S. pneumoniae* in their nasopharynx asymptomatically pose a significant risk for community-acquired pneumonia infections, particularly when they are exposed to factors that weaken the immune system. In present study, the overall prevalence of *S. pneumoniae* carriage rate among primary school children was 16% (95% CI: 12.0–20.0). This finding is comparable with the studies conducted in Ethiopia (12%), [31], (18.4%) [32], Iran (13.9%) [33], and Italy (14.9%) [34]. The findings of the present study are lower than the report from Ethiopia (43.8%) [23], (42.6%) [10], Turkey (21.9%) [30] and Indonesia (49.5%) [35]. The possible explanation for the difference in the carriage rate might be due to socioeconomic status, study setting, sample size and season [36–39]. However, our result is higher than previous reports from China (3.5%) [40]. The possible reasons for the variation might be do to age differences, methodology, and media used [11,36,38].

In this study, children who had been living together with two or more than two siblings less than five years old in the house were four times more likely to be colonized with *S. pneumoniae* than those who did not have any sibling(s) less than five years old in the house. This finding is in agreement with the results reported in Hawassa [10], Gonder [41], and Jimma Ethiopia [42]. This might be because the younger siblings are more susceptible to carrying *S. pneumoniae* due to their immature immune system exposure [43]. Younger siblings are often near each other, leading to increased contact which facilitates the transmission of *S. pneumoniae*, especially in households with multiple young children [44].

In our study being a passive smoker appeared to be associated with *S. pneumoniae* carriage [45]. Children living in the family of cigarette smokers were two times at risk of being *S. pneumoniae* carriers in their nasopharynx compared to children living in the family of non-cigarette smokers. This finding is concordant with the results reported from Ethiopia [46] and Iran [33]. Passive smoke exposure disrupts the normal function of the respiratory tract’s mucociliary clearance system, allowing *S. pneumoniae* to persist in the nasopharynx [47,48]. Passive smoking weakens the immune system of exposed Children and allows *S. pneumoniae* to establish and persist in the nasopharynx [17,49].

In the present study, the children living in a house having one room were two times at risk of being *S. pneumoniae* carriers. This result is in line with the reports from Ethiopia [11,41,46]. The possible reasons for this could be close contact, poor ventilation, increased exposure, poor hygiene practices, and transmission dynamics [50]. In a single-room house, family members are often overcrowded, leading to increased contact with each other. Poor ventilation can result in stagnant air, which increases the concentration of respiratory droplets containing *S. pneumoniae*. Children living in single-room houses shared sleeping spaces, common areas, and limited personal space which contribute to direct and indirect exposure to *S. pneumoniae* [50]. Poor hygiene increases the risk of *S. pneumoniae* colonization in the nasopharynx due to limited access to handwashing facilities and personal space resulting in inadequate hygiene practices. Children in single-room houses are more likely to share utensils, towels, and other items, leading to person-to-person transmission within families [38,51].

In this study, those children who had a history of previous upper respiratory infections were three times more likely to be *S. pneumoniae* carriers compared to those who had no history of upper respiratory infections. This finding is comparable with the result reported from Ethiopia [46]. The possible explanation for this might be due to increased risk after URTI, Immune response, and susceptibility [52]. URTIs such as influenza, common colds, sinusitis, and pharyngitis can weaken the mucosal barrier, making it easier for *S. pneumoniae* to establish itself and colonize the nasopharynx [53]. Children recovering from URTI may have transient immunosuppression, rendering them more susceptible to *S. pneumoniae* colonization [54].

In this study, the results of the antimicrobial susceptibility test showed that about 34(62.96%), 38(70.37%), 44(81.48%), 39(72.2%), 41(75.9%) and 34(62.96%) of isolated *S. pneumoniae* were susceptible to chloramphenicol, clindamycin, erythromycin, oxacillin, vancomycin and TMP-SMX respectively. In this study about (14.8%) of *S. pneumoniae* were resistant to erythromycin which is comparable to the study done in Hawassa(12.9%) and Harar(11.6%), Ethiopia) [55]. In this study, the isolated *S. pneumoniae* were more resistant to Tetracycline(42.6%) and trimethoprim-sulfamethoxazole (33.3%) than all other five antibiotics. The result of Tetracycline(42.6%) in our study was in line with the findings reported from Ethiopia, in Hawassa(37.3%) [19] and in Harar(41.8%) [56], lower than in Jimma(53.2%) [42], Gonder(68.8%) [46] and Jordan(53.8%) [57]. However, higher than the finding reported in Spain (26.7%) [13].

In present study, the results of Trimethoprimsulfamethoxazole(33.3%) was consistent to the findings recorded in Ethiopa, Hawassa(34.2%) [19], Jimma (38%) [42,58], lower than the study conducted in Jordan, which found (73.8%) [57] and Ethiopia, Harar (46.5%). However, our finding is higher than the results reported in Spain(28.2%) [13] and in Ethiopia, Addis Ababa(24.6%) [58]. The possible explanation for this resistance variation could be due to unnecessarily use of antimicrobial drugs, incorrectly prescribing of antibiotics and inappropriate antibiotics consumption(patients not following the prescribed dose or stopping treatment [59,60].

### Limitation of the study

This study has notable limitations that affect the interpretation of its findings. A key limitation is the lack of serotyping assessment and molecular characterization of isolated bacterial agents. While culturing, biochemical tests, and antimicrobial susceptibility tests were conducted, these methods may miss non-culturable bacteria and do not provide strain-level identification or reveal genetic mechanisms of resistance. Additionally, potential recall bias in self-reported health histories poses another challenge. Participants’ inaccuracies in recalling their health status, prior illnesses, and antibiotic usage could lead to inconsistencies in the data, skewing results.

## Conclusions and recommendations

### Conclusions

The study revealed an asymptomatic carriage rate of Streptococcus pneumoniae at 16% among the population studied. This finding indicates a significant presence of the bacteria in the nasopharynx, which can contribute to the transmission of respiratory infections. Notably, the isolates exhibited a concerning level of antibiotic resistance, with tetracycline resistance at 42.6% and TMP-SMX resistance at 33.3%. These high resistance rates highlight the challenges in treating infections caused by S. pneumoniae and raise concerns about the effectiveness of common antibiotics. In contrast, the study identified erythromycin and vancomycin as the most effective drugs against the isolates. This information is crucial for guiding treatment strategies in clinical settings, particularly in regions where antibiotic resistance is prevalent. The study also identified several associated risk factors for nasopharyngeal carriage of S. pneumoniae. These include having two or more siblings under the age of five, being a passive smoker, living in a single-room household, and having a previous history of respiratory tract infections. These factors can contribute to increased transmission and carriage rates, particularly in crowded or poorly ventilated living environments.

### Recommendations

To address the issue of S. pneumoniae nasopharyngeal carriage among schoolchildren in Bisidimo, Ethiopia, several recommendations can be made:

- Health Education: Implement health education programs specifically targeting lower-level education students. These programs should focus on the identified risk factors, teaching children and their families about the importance of hygiene, respiratory health, and the implications of passive smoking.
- Regular Screening Programs: Establish regular screening initiatives to monitor S. pneumoniae carriage rates in schools and communities. Early identification of carriers can help in implementing timely interventions to prevent the spread of infections.
- Vaccination Initiatives: Promote vaccination against S. pneumoniae within the community, particularly for children under five years old, as they are at higher risk. Ensuring access to vaccines can significantly reduce the incidence of pneumococcal diseases.
- Community Education on Hygiene: Conduct community-wide education campaigns emphasizing the importance of hygiene practices, such as handwashing and respiratory etiquette, to reduce the transmission of respiratory pathogens.
- Antibiotic Stewardship Programs: Develop and implement antibiotic stewardship programs aimed at educating healthcare providers and the community about the responsible use of antibiotics. This can help mitigate the impact of antibiotic resistance.
- Further Research: Encourage further research into local risk factors contributing to S. pneumoniae carriage and resistance patterns. Enhanced surveillance can provide valuable data for public health strategies and inform future interventions.

## Supporting information

S1 Data Supporting information SPSS data. (XLSX)
